# Association of Gestational Age at Birth With Subsequent Suspected Developmental Coordination Disorder in Early Childhood in China

**DOI:** 10.1001/jamanetworkopen.2021.37581

**Published:** 2021-12-14

**Authors:** Jing Hua, Anna L. Barnett, Gareth J. Williams, Xiaotian Dai, Yuanjie Sun, Haifeng Li, Guixia Chen, Lei Wang, Junyan Feng, Yingchun Liu, Lan Zhang, Ling Zhu, Tingting Weng, Hongyan Guan, Yue Gu, Yingchun Zhou, Andrew Butcher, Wenchong Du

**Affiliations:** 1 Shanghai First Maternity and Infant Hospital, Tongji University School of Medicine, Shanghai, China; 2 Centre for Psychological Research, Oxford Brookes University, Oxford, United Kingdom; 3 School of Social Sciences, Nottingham Trent University, Nottingham, United Kingdom; 4 Department of Rehabilitation, The Children's Hospital, Zhejiang University School of Medicine, National Clinical Research Center for Child Health, Zhejiang, China; 5 Department of Children Healthcare, Women and Children’s Hospital, School of Medicine, Xiamen University, Fujian, China; 6 Department of Child Health Care, Maternal and Child Health Care Hospital of Yangzhou, Affiliated Hospital of Medical College Yangzhou University, Jiangsu, China; 7 Department of Developmental Behaviour Pediatrics, The First Hospital of Jilin University, Jilin, China; 8 Maternity Service Center of Changchun Maternal & Child Health Care Hospital, Jilin, China; 9 Chengdu Women's and Children's Central Hospital, School of Medicine, University of Electronic Science and Technology of China, Sichuan, China; 10 Maternal and Child Health Hospital of Shanxi, Shanxi, China; 11 Maanshan Maternal and Child Health Hospital of Anhui Province, Anhui, China; 12 Capital Institute of Pediatrics, Beijing, China; 13 School of Statistics, East China Normal University, Shanghai, China; 14 Department of Psychology, Nottingham Trent University, Nottingham, United Kingdom

## Abstract

**Importance:**

It remains unknown whether children born at different degrees of prematurity, early term, and post term might have a higher risk of developmental coordination disorder (DCD) compared with completely full-term children (39-40 gestational weeks).

**Objective:**

To differentiate between suspected DCD in children with different gestational ages based on a national representative sample in China.

**Design, Setting, and Participants:**

A retrospective cohort study was conducted in China from April 1, 2018, to December 31, 2019. A total of 152 433 children aged 3 to 5 years from 2403 public kindergartens in 551 cities of China were included in the final analysis. A multilevel regression model was developed to determine the strength of association for different gestational ages associated with suspected DCD when considering kindergartens as clusters.

**Main Outcomes and Measures:**

Children’s motor performance was assessed using the Little Developmental Coordination Disorder Questionnaire, completed by their parents. Gestational age was determined according to the mother’s medical records and divided into 7 categories: completely full term (39 to 40 weeks’ gestation), very preterm (<32 weeks), moderately preterm (32-33 weeks), late preterm (34-36 weeks), early term (37-38 weeks), late term (41 weeks), and post term (>41 weeks).

**Results:**

A total of 152 433 children aged 3 to 5 years (mean [SD] age, 4.5 [0.8] years), including 80 370 boys (52.7%) and 72 063 girls (47.3%), were included in the study. There were 45 052 children (29.6%) aged 3 years, 59 796 (39.2%) aged 4 years, and 47 585 (31.2%) aged 5 years. Children who were born very preterm (odds ratio [OR], 1.35; 95% CI, 1.23-1.48), moderately preterm (OR, 1.18; 95% CI, 1.02-1.36), late preterm (OR, 1.24; 95% CI, 1.16-1.32), early term (OR, 1.11; 95% CI, 1.06-1.16), and post term (OR, 1.17; 95% CI, 1.07-1.27) were more likely to be classified in the suspected DCD category on the Little Developmental Coordination Disorder Questionnaire than completely full-term children after adjusting for the same characteristics. Additionally, there was no association with suspected DCD in younger (aged 3 years) early-term and postterm children by stratified analyses.

**Conclusions and Relevance:**

In this cohort study, every degree of prematurity at birth, early-term birth, and postterm birth were associated with suspected DCD when compared with full-term birth. These findings have important implications for understanding motor development in children born at different gestational ages. Long-term follow-up and rehabilitation interventions should be considered for children born early and post term.

## Key Points

**Question:**

In addition to preterm birth, are early-term and postterm birth also associated with suspected developmental coordination disorder in early childhood when compared with full-term birth?

**Findings:**

In this cohort study of 152 433 children aged 3 to 5 years in China, early and postterm birth were associated with impaired motor performance compared with completely full-term birth when adjusting for kindergartens (as clusters) and child, family, and maternal health characteristics.

**Meaning:**

These findings suggest that long-term follow-up and rehabilitation interventions should be considered in children born early and post term.

## Teaser

This cohort study investigates the association of gestational age at birth with suspected developmental coordination disorder in early childhood among children aged 3 to 5 years in China.

## Introduction

Developmental coordination disorder (DCD) is characterized by marked impairment of motor coordination, which commonly results in persistent and significant difficulties in performing daily activities involving balance or manual skills.^1,2,3^ The prevalence of DCD in children aged 5 to 11 years is estimated to be 5% to 6% worldwide,^4^ with an even higher rate of prevalence (8.3%) in China.^5,6^ However, due to the large variability in normal motor development, a diagnosis of DCD is generally not recommended before the age of 5 years.^7^

Studies investigating the risk factors for DCD suggest that prenatal and perinatal influences may be associated with the development of later impairments.^5^ Preterm infants are at a significantly higher risk of suboptimal brain development,^8^ with the risk of DCD increasing with younger gestational age.^9,10^ Children born very preterm (<32 weeks) have been found to have a higher risk of developing DCD.^11,12,13,14,15,16^ Mild and moderate motor impairments occur in almost half of all preterm children (<37 weeks).^17^ However, although it has been reported that late preterm children (34-36 weeks) have more neonatal morbidities than full-term infants^18^ and experience neuromotor delay during their first year of life,^19^ few studies have explored the association between late preterm birth and DCD in preschool or school-aged children beyond the first year of life. There appear to be inconsistencies in the literature, with studies showing that late preterm infants otherwise born healthy seem to have no delay in their cognition, motor performance, behavior, or socioemotional development throughout childhood,^20,21^ whereas other studies report significant differences in neurodevelopment between late preterm and full-term children.^21,22,23,24^

Gestational age is a crucial factor in predicting motor development and DCD,^25^ but less attention has been paid to the relationship between gestational age in weeks and motor development in children born full term (≥37 weeks). Existing data have indicated that longer gestation (within 37-41 weeks) is associated with better cognitive and psychomotor development in children aged 12 months.^26^ Other studies have reported that children born at 39 weeks or later have fewer neonatal morbidities^27^ and better cognitive and academic outcomes than those born at 37 to 38 weeks (early term but still full-term birth).^26,28,29,30,31^ The number of gestational weeks in the full-term range has also been found to be associated with neuromotor and motor development in infants aged 9 to 15 weeks^32^ and infants aged 12 months.^26^

Recently, studies^33,34,35,36,37^ have reported that postterm birth (>41 weeks) is negatively associated with a child’s short-term and long-term health outcomes. A meta-analysis^38^ found that postterm birth is associated with significant negative effects on cognitive measures compared with full-term birth. Children born post term were more likely to have emotional and behavioral problems at both age 18 and 36 months compared with full-term children.^36^ Postterm children were also found to manifest increased risk and symptomatology of autism spectrum disorder^33,39^ and attention-deficit/hyperactivity disorder.^40^ However, children born post term were also reported to reach the main developmental milestones in their infancy when compared with full-term children.^41^ Therefore, the literature on the association between postterm birth and later development remains inconclusive.

In this study, we used a retrospective cohort design to examine the association of gestational age with suspected DCD within a sample of urban Chinese children. We hypothesized that children born at every degree of prematurity (<37 weeks), early term (37-38 weeks), and post term (>41 weeks) had an increased risk of suspected DCD compared with children born completely full term (39-40 weeks). The aims of this study were to (1) investigate the associated risk of suspected DCD in children born at different gestational weeks and (2) explore the association between gestational age and the risk of suspected DCD by age and sex.

## Methods

### Study Population and Study Design

The present study was part of a large national retrospective cohort study in China to explore neurobehavioral development in Chinese children. A stratified cluster sampling plan was used to ensure that the participants included in the current study were representative of the Chinese population. China's 2018 to 2019 National Census data provided the basis for the stratification by geographic region, age, sex, and socioeconomic status. Ethnic information was not collected because more than 99% of the population in the targeted regions were Han according to the National Census. The government-supported maternity and children’s health center in each city was selected to invite their local kindergartens to participate in the study. Class teachers were responsible for distributing the notification to parents to complete the online questionnaire; names and phone numbers of the researchers were provided in case the parents had queries. We used an electronic questionnaire system to enhance the quality of the data by allowing the inclusion of pop-up instructions, error messages, links to further information, and to set conditions to ensure participants could not skip questions. A data coordination center was established to take charge of establishing, managing, and maintaining the database and website, coordinating among health centers.

Only mainstream schools and nurseries were included in the study. Children with severe visual, hearing, or intellectual impairments (according to the examinations before starting kindergarten) or other severe developmental disorders who were required to attend special education schools or nurseries according to the local regulations were excluded. From April 1, 2018, to December 31, 2019, a total of 189 375 preschoolers were recruited from 2403 mainstream kindergartens in 551 cities of China.

**Figure 1.**
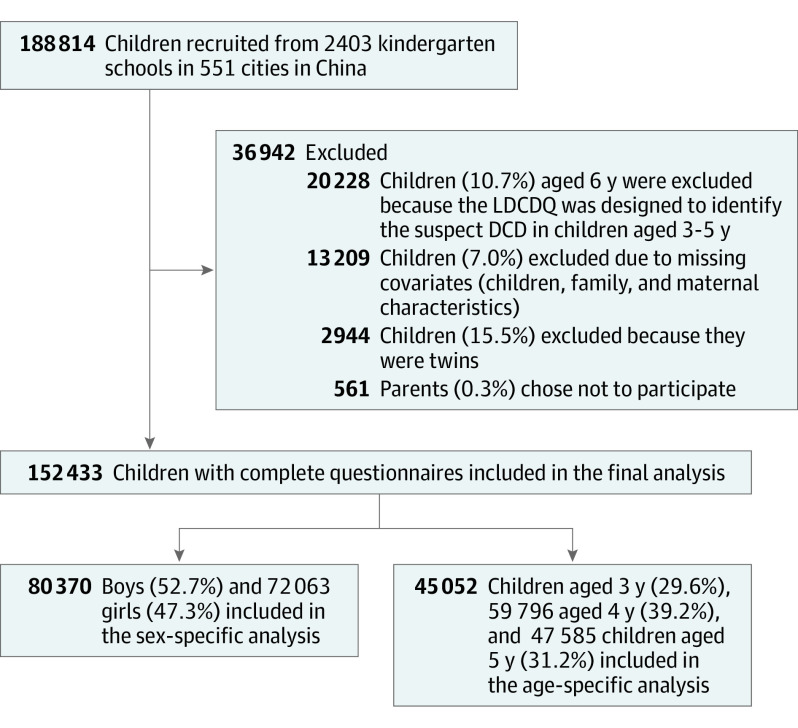
Flowchart of the Study Population DCD indicates developmental coordination disorder; LDCDQ, Little Developmental Coordination Disorder Questionnaire.

It is a normal practice for parents to keep in touch with their children’s nursery via smart devices in China, including all of the kindergartens involved in the current study. It was therefore assumed that all of the parents in the current study had relatively high proficiency in online questionnaire completion. A very small proportion of the parents (n = 561; 0.3%) chose not to participate or left the questionnaire before fully completing it. Children aged 6 years or those with missing covariates were also excluded, leaving a total of 152 433 children for the final analysis (Figure 1).

The study was approved by the ethics committee of Shanghai First Maternity and Infant Hospital (KS18156). The parents had given online written consent to participate in the study before completing the online questionnaire. This study followed the Strengthening the Reporting of Observational Studies in Epidemiology (STROBE) reporting guideline.

### Outcomes, Independent Variables, and Other Covariates

Children’s motor performance was assessed using the Little Developmental Coordination Disorder Questionnaire (LDCDQ). The LDCDQ was developed to screen for motor coordination difficulties in children aged 3 and 4 years,^42^ and it can also be extended for use with children as old as 5 years.^43^ The LDCDQ consists of 15 items divided into 3 subcategories: control during movement, fine motor skills, and general coordination. Each category contains 5 items; for each item, parents are asked to compare the performance of their child with that of children of the same age and sex and to rate their child’s performance on a 5-point Likert scale (1 point, not at all relevant to my child, to 5 points, extremely relevant to my child). Each subcategory has a maximum score of 25 points. Scores are summed to give a maximum total score of 75 points, with higher scores indicating a higher level of motor proficiency. A previous study^44^ reported that the Chinese version of the LDCDQ has high internal consistency and split-half reliability and fair factor construct validity. According to previous recommendations,^43,45^ we used the age- and sex-specific norms of the LDCDQ, and cutoff scores were provided based on a national sample in China to indicate suspected impairments of motor coordination (suspected DCD was defined as LDCDQ ≤15th percentile; probably not DCD was defined as LDCDQ >15th percentile).

### Independent Variables

Gestational age at birth was obtained from the mother’s medical records and was based on ultrasonography examination and date of last menstrual period. Gestational weeks were divided into 7 categories^40,46^: completely full term was defined as 39 to 40 weeks’ gestation, and the other 6 categories included very preterm (<32 weeks), moderately preterm (32-33 weeks), late preterm (34-36 weeks), early term (37-38 weeks), late term (41 weeks), and post term (>41 weeks).

### Covariates

We included the child, family, and maternal health characteristics as potential confounders when exploring the association between gestational age and suspected DCD (Table 1). Most of these variables were dichotomized into yes or no; body mass index (calculated as weight in kilograms divided by height in meters squared) was used as an indicator of obesity based on each child’s height and weight.^47^ Family structures were classified into 3 types: 3-generation (or more) family, nuclear family, and single-parent family. The 3-generation (or more) family refers to a child who lives with their parents and grandparents, which is a traditional family structure in China; a nuclear family refers to a child who lives with only their parents; and single-parent family means that the child lives with 1 of their parents. We divided maternal age into 3 age bands: younger than 30 years, 30 to 34 years, and older than 34 years.^48^ Maternal complications of pregnancy and delivery were defined according to the *International Statistical Classification of Diseases and Related Health Problems, Tenth Revision*. The classification is defined as having 1 of the following maternal complications during pregnancy: vaginal bleeding during pregnancy, risk of miscarriage, use of antibiotics, use of fertility drugs, intrauterine distress, or fetal asphyxia.

**Table 1.** Child, Family, and Maternal Health Characteristics During Pregnancy in the Study Population (N = 152 433)

Characteristic	Total	No. (%)^a^				
		Sex		Age, y		
		Male	Female	3	4	5
**Child characteristics**						
Children’s age, mean (SD), y	4.5 (0.8)	4.5 (0.8)	4.5 (0.8)	3.5 (0.3)	4.4 (0.3)	5.4 (0.3)
BMI, mean (SD)	15.6 (1.6)	15.7 (1.6)	15.4 (1.6)	15.8 (1.4)	15.5 (1.6)	15.5 (1.8)
Right-handedness						
No	11 290 (7.4)	6560 (8.2)	4730 (6.6)	4137 (9.2)	4439 (7.5)	2714 (5.7)
Yes	141 143 (92.6)	73 810 (91.8)	67 333 (93.4)	40 915 (90.8)	55 357 (92.5)	44 871 (94.3)
Eyesight^b^						
Normal	137 378 (90.1)	72 371 (90.1)	65 007 (90.2)	40 862 (90.7)	53 995 (90.3)	42 521 (89.4)
Abnormal	15 055 (9.9)	7999 (9.9)	7056 (9.8)	4190 (9.3)	5801 (9.7)	5064 (10.6)
Birth weight, g						
<2500	6303 (4.1)	3068 (3.8)	3235 (4.5)	1724 (3.8)	2659 (4.5)	1920 (4.0)
≥2500	146 130 (95.9)	77 302 (96.2)	68 828 (95.5)	43 328 (96.2)	57 137 (95.5)	45 665 (96.0)
Gestational age at birth, wk						
<32 (very preterm)	5439 (3.6)	2930 (3.6)	2509 (3.5)	1384 (3.1)	2100 (3.5)	1955 (4.1)
32-33 (moderately preterm)	2322 (1.5)	1255 (1.6)	1067 (1.5)	589 (1.3)	966 (1.6)	767 (1.6)
34-36 (late preterm)	12 915 (8.5)	7146 (8.9)	5769 (8.0)	3463 (7.7)	5024 (8.4)	4428 (9.3)
37-38 (early term)	38 875 (25.5)	21 484 (26.7)	17 391 (24.1)	11 609 (25.8)	15 069 (25.2)	12 197 (25.6)
39-40 (completely full term)	76 501 (50.2)	39 448 (49.1)	37 053 (51.4)	23 180 (51.5)	30 142 (50.4)	23 179 (48.7)
41 (late term)	8923 (5.8)	4322 (5.4)	4591 (6.4)	2843 (6.3)	3542 (5.9)	2538 (5.4)
>41 (post term)	7458 (4.9)	3775 (4.7)	3683 (5.1)	1984 (4.4)	2953 (5.0)	2521 (5.3)
Mode of delivery						
Vaginal	81 718 (53.6)	42 209 (52.5)	39 509 (54.8)	24 766 (55.0)	32 136 (53.7)	24 816 (52.2)
Cesarean	70 715 (46.4)	38 161 (47.4)	32 554 (45.2)	20 286 (45.0)	27 660 (46.3)	22 769 (47.8)
NICU admission						
No	137 063 (89.9)	71 690 (89.2)	65 373 (90.7)	40 137 (89.1)	53 816 (90.0)	43 110 (90.6)
Yes	15 370 (10.1)	8680 (10.8)	6690 (9.3)	4915 (10.9)	5980 (10.0)	4475 (9.4)
Other developmental disorders^c^						
No	151 474 (99.4)	79 791 (99.3)	71 683 (99.5)	44 802 (99.5)	59 426 (99.4)	47 246 (99.3)
Yes	959 (0.6)	579 (0.7)	380 (0.5)	250 (0.5)	370 (0.6)	339 (0.7)
Psychiatric medication						
No	151 418 (99.3)	79 778 (99.3)	71 640 (99.4)	44 694 (99.2)	59 409 (99.3)	47 315 (99.4)
Yes	1015 (0.7)	592 (0.7)	423 (0.6)	358 (0.8)	387 (0.7)	270 (0.6)
**Family characteristics**						
Higher education of mother^d^						
No	70 473 (46.2)	37 710 (46.9)	32 763 (45.5)	19 149 (42.5)	27 165 (45.4)	23 426 (49.2)
Yes	81 960 (53.8)	42 660 (53.1)	39 300 (54.5)	25 903 (57.5)	32 631 (54.6)	24 159 (50.8)
Higher education of father^d^						
No	71 493 (46.9)	38 184 (47.5)	33 309 (46.2)	19 816 (44.0)	27 452 (45.9)	24 225 (50.9)
Yes	80 940 (53.1)	42 186 (52.5)	38 754 (53.8)	25 236 (56.0)	32 344 (54.1)	23 360 (49.1)
Mother’s occupation						
Employed	95 328 (62.5)	50 580 (62.9)	44 748 (62.1)	28 643 (63.6)	37 398 (62.5)	29 287 (61.5)
Unemployed	57 105 (37.5)	29 790 (37.1)	27 315 (37.9)	16 409 (36.4)	22 398 (37.5)	18 298 (38.5)
Father’s occupation						
Employed	120 489 (79.0)	63 548 (79.1)	56 941 (79.0)	36 305 (80.6)	47 258 (79.0)	36 926 (77.6)
Unemployed	31 944 (21.0)	16 822 (21.0)	15 122 (21.0)	8747 (19.4)	12 538 (21.0)	10 659 (22.4)
Family annual per-capita income, RMB^e^						
<30 000	28 419 (18.6)	14 829 (18.5)	13 590 (18.9)	7839 (17.4)	11 277 (18.9)	9303 (19.6)
≥30 000	124 014 (81.4)	65 541 (81.5)	58 473 (81.1)	37 213 (82.6)	48 519 (81.1)	38 282 (80.5)
Family structure						
Single-parent families	3610 (2.4)	1808 (2.3)	1802 (2.5)	899 (20.0)	1410 (2.4)	1301 (2.7)
Nuclear families	94 990 (62.3)	50 269 (62.6)	44 721 (62.1)	26 528 (58.9)	37 390 (62.5)	31 072 (65.3)
Extended families	53 833 (35.3)	28 293 (35.2)	25 540 (35.4)	17 625 (39.1)	20 996 (35.1)	15 212 (32.0)
No. of children in the family						
1	82 230 (54.0)	44 355 (55.2)	37 875 (52.6)	24 917 (55.3)	322 595 (54.5)	24 718 (51.9)
≥2	70 203 (46.0)	36 015 (44.8)	34 188 (47.4)	20 135 (44.7)	27 201 (45.5)	22 867 (48.1)
Maternal health during pregnancy						
Maternal age at delivery, y						
<30	115 739 (75.9)	61 024 (75.9)	54 715 (75.9)	33 061 (73.4)	45 260 (75.7)	37 418 (78.6)
30-34	27 497 (18.1)	14 553 (18.1)	12 944 (18.0)	8811 (19.5)	10 957 (18.3)	7729 (16.3)
≥35	9197 (6.0)	4793 (6.0)	4404 (6.1)	3180 (7.1)	3579 (6.0)	2438 (5.1)
Smoking or passive smoking during pregnancy						
No	111 163 (72.9)	58 631 (73.0)	52 532 (72.9)	32 748 (72.7)	43 546 (72.8)	34 869 (73.3)
Yes	41 270 (27.1)	21 739 (27.0)	19 531 (27.1)	12 304 (27.3)	16 250 (27.2)	12 716 (26.7)
Maternal complications during pregnancy^f^						
No	145 088 (95.2)	76 567 (95.3)	68 521 (95.1)	42 517 (94.4)	57 032 (95.4)	45 539 (95.7)
Yes	7345 (4.8)	3803 (4.7)	3542 (4.9)	2535 (5.6)	2764 (4.6)	2046 (4.3)

Abbreviations: BMI, body mass index (calculated as weight in kilograms divided by height in meters squared); NICU, neonatal intensive care unit; RMB, renminbi (Chinese currency).

^a^ Units written as No. (%) unless otherwise specified.

^b^ Eyesight indicates the power or faculty of seeing.

^c^ Other developmental disorders included autism spectrum disorder, attention-deficit/hyperactivity disorder, and learning disorders.

^d^ Higher education of mother or father indicates tertiary education leading to an academic degree.

^e^ The national average family per-capita income of the year before the survey time. As of November 12, 2021, 1 RMB is equal to 0.16 US dollars.

^f^ Indicates having 1 of the following maternal complications during pregnancy: vaginal bleeding during pregnancy, risk of miscarriage, use of antibiotics, use of fertility drugs, intrauterine distress, or fetal asphyxia.

### Statistical Analysis

A mixed model using a random intercept (we hypothesized that there was no interaction between kindergartens and total LDCDQ scores) was used to investigate the associations of the different gestational ages (very preterm, moderately preterm, late preterm, early term, late term, and post term) with the total score and subscores of LDCDQ compared with full-term birth. A multilevel logistic regression model was used to determine the strength of association for different gestational ages associated with suspected DCD. In the mixed and logistic regression models, we considered the kindergartens as primary sampling units and other potential confounders (child, family, and maternal health characteristics as shown in Table 1). All *P* values were 2-sided, and *P* < .05 was denoted as statistically significant. Statistical analyses were performed using the lmer and Glmer functions in R, version 4.0.1 (R Core Team).

## Results

A total of 152 433 children aged 3 to 5 years old (mean [SD] age, 4.5 [0.8] years; 80 370 boys [52.7%]; 72 063 girls [47.3%]) were included in the study. There were 45 052 children (29.6%) aged 3 years, 59 796 (39.2%) aged 4 years, and 47 585 (31.2%) aged 5 years. A total of 5439 births (3.6%) were very preterm, 2322 (1.5%) were moderately preterm, 12 915 (8.5%) were late preterm, 38 875 (25.5%) were early term, 76 501 (50.2%) were full term, 8923 (5.9%) were late term, and 7458 (4.9%) were post term. The child, family, and maternal health during pregnancy characteristics in the study population are shown in Table 1. The LDCDQ scores and rates of suspected DCD by different gestational weeks are shown in eTable 1 in the Supplement and Table 2.

**Table 2.** Association Between Gestational Age and Suspected DCD in Preschoolers (N = 152 433)

Gestational age	Probably not DCD, No. (%) (n = 128 106)	Suspected DCD, No. (%) (n = 24 327)	Suspected DCD vs probably not DCD			
			Crude		Adjusted^a^	
			OR (95% CI)	*P* value	OR (95% CI)	*P* value
Very preterm (<32 wk)	4171 (76.7)	1268 (23.3)	1.70 (1.59-1.81)	<.001	1.52 (1.42-1.63)	<.001
Moderately preterm (32-33 wk)	1841 (79.3)	481 (20.7)	1.49 (1.35-1.66)		1.349 (1.21-1.50)	
Late preterm (34-36 wk)	10 338 (80.1)	2577 (20.0)	1.41 (1.34-1.48)		1.29 (1.23-1.36)	
Early term (37-38 wk)	32 531 (83.7)	6344 (16.3)	1.14 (1.10-1.18)		1.11 (1.07-1.15)	
Completely full term (39-40 wk)	65 430 (85.5)	11 071 (14.5)	1 [Reference]		1 [Reference]	
Late term (41 wk)	7658 (85.8)	1265 (14.2)	0.98 (0.92-1.04)		0.98 (0.91-1.04)	
Post term (>41 wk)	6137 (82.3)	1321 (17.7)	1.23 (1.15-1.31)		1.15 (1.08-1.23)	

Abbreviations: DCD, developmental coordination disorder; OR, odds ratio.

^a^ Adjusted for kindergartens (as cluster to control the unmeasured factors in kindergarten environment), child, family, and maternal health characteristics during pregnancy.

When children in the suspected DCD and probably not DCD groups for each gestational age category were compared with the completely full-term category, adjusting for kindergarten, child, family, and maternal health characteristics, children with very preterm (odds ratio [OR] 1.52; 95% CI, 1.42-1.63; *P* < .001), moderately preterm (OR, 1.35; 95% CI, 1.21-1.50; *P* < .001), late preterm (OR, 1.29; 95% CI, 1.23-1.36; *P* < .001), early term (OR, 1.11; 95% CI, 1.07-1.15; *P* < .001), and postterm births (OR, 1.15; 95% CI, 1.08-1.23; *P* < .001) had increased risk of suspected DCD. Late term birth was not associated with an increased risk of suspected DCD (Table 2). Children who were born very preterm (OR, 1.35; 95% CI, 1.23-1.48), moderately preterm (OR, 1.18; 95% CI, 1.02-1.36), late preterm (OR, 1.24; 95% CI, 1.16-1.32), early term (OR, 1.11; 95% CI, 1.06-1.16), and post term (OR, 1.17; 95% CI, 1.07-1.27) were more likely to be classified in the suspected DCD category on the LDCDQ than completely full-term children after adjusting for the same characteristics (Table 2). Additionally, the total LDCDQ scores and subscores (motor control, writing/fine motor skills, and general coordination) were significantly lower for each gestational age category when compared with the completely full-term category when adjusting for the same covariates (eTable 2 in the Supplement). However, there were no significant differences in the suspected DCD and LDCDQ score between late-term birth and completely full-term birth.

**Figure 2.**
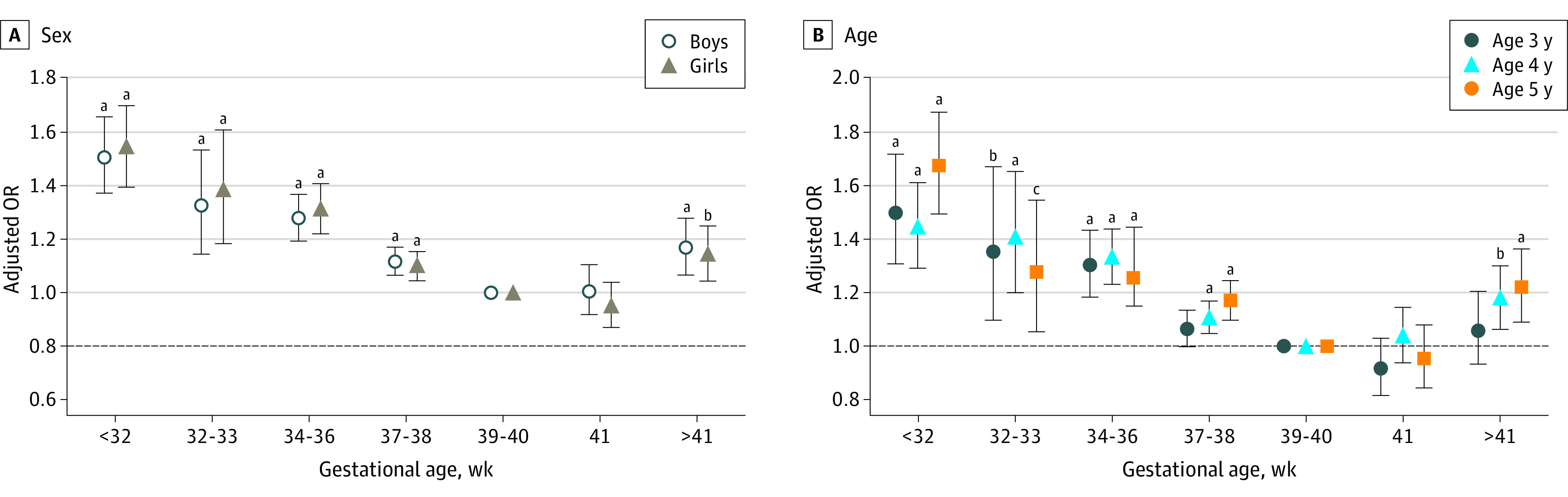
Association Between Gestational Age and Risk of Suspected Developmental Coordination Disorder in Preschoolers When Adjusting for Child, Family Characteristic, and Maternal Health by Sex and Age (N = 152 433) Error bars indicate 95% CIs. OR indicates odds ratio. ^a^*P* < .05. ^b^*P* < .01. ^c^*P* < .001.

When we divided the children into age years, the results showed that the statistically significant association between gestational age and LDCDQ total score remained in the preterm and early-term children; however, there was no association in postterm children aged 3 years (eFigure in the Supplement). There was no association between gestational age and risk of suspected DCD in early-term and postterm children aged 3 years (Figure 2). The adjusted associations of gestational age (including preterm, early term, and post term) with the LDCDQ total score (eFigure in the Supplement) and with suspected DCD (Figure 2) remained significant in both boys and girls.

## Discussion

To our knowledge, this is the first study with a large national representative population sample to explore the association between gestational age in a full range and suspected DCD. We found significant associations in every degree of prematurity with suspected DCD. More importantly, associations between early term (37-38 weeks) and postterm (>41 weeks) birth and suspected DCD were also found when compared with full-term birth. Additionally, we observed that these associations did not exist in younger children (aged 3 years) in stratified analyses.

Our findings were consistent with previous work, which also found that DCD was more likely to occur in children born preterm, especially in those born very preterm^11,15,16,49,50^ and moderately preterm.^9^ Infants who were born both moderately preterm and early term showed a higher level of impairment in fine motor skills than infants born full term.^51^ Infants with moderate and late preterm births displayed a neuromotor delay during the first year of life.^19^ Previous studies suggested that the brain microstructure is highly correlated with motor impairment in preterm children. For instance, white matter alterations were found in children born preterm with very low birth weight.^52^ Higher fractional anisotropy in all major white matter tracts in very preterm infants at term-equivalent age was found to be correlated with a superior fine motor performance at age 2 years.^53^ Additionally, brain development occurs in a very specific order and time frame.^29^ Preterm infants cared for in neonatal intensive care units (NICUs) face substantial developmental challenges that differ substantially from the natural brain maturation that normally occurs in the uterus. The varying clinical processes in the NICU have different effects on the shape of the brainstem, as is evident in several distinct neurodevelopmental profiles observed in premature newborns.^54^ Studies have also shown that morphine exposure and exposure to painful procedures were associated with poor cerebellar growth in the neonatal period and neurodevelopmental impairment in very preterm children.^55,56^ Moreover, the brain structures of early term and full-term infants were found to differ in their concurrent motor, neurologic, and neurobehavioral functions when compared with those of a full-term infant.^57^

We also found decreased motor performance and increased risk of suspected DCD in early-term children (37-38 weeks) after accounting for a broad range of possible confounders including child, family, and maternal health characteristics. Previous studies emphasized the importance of the last few weeks during gestation, when a large portion of brain development takes place.^12^ The volume of total gray matter increases by approximately 1.4% per week from 29 to 41 weeks of gestation, along with a 5-fold increase in white matter volume between 35 and 41 weeks of gestation.^58,59^ The external granular layer expands horizontally to accommodate a more than 30-fold increase in the hemisphere surface area that occurs from 30 to 40 gestational weeks; therefore, the number of cells increases substantially.^60^ Neuroimaging research indicates that longer gestation is associated with region-specific increases in gray matter density^61^ and a more efficient neural network.^62^ Not only preterm birth, but also early term birth, can cause disruption at specific intervals during the brain’s development of neural connections for specific cognitive areas.^63^ A recent systematic review reported that children born early term are at an increased risk of having cognitive deficits, poorer school performance, and behavioral problems compared with children born full term.^64^ To our knowledge, our study is the first to report an association between early term birth and an increased risk of suspected DCD, which suggests that children born early term should be monitored more carefully owing to the wide range of developmental effects.

Another finding of our study was the observed association between postterm birth and suspected DCD. It has been previously reported that postterm birth increases the risks of cognitive impairments, severe mental disorders, autism spectrum disorder, attention-deficit/hyperactivity disorder, and other behavioral and emotional problems in early childhood.^33,34,38,39,65,66^ Our study results suggest that children born after 41 gestational weeks were also more likely to develop DCD. This may have been due to the complicated conditions related to postterm birth, including prolonged labor, cephalopelvic disproportion, and shoulder dystocia,^67^ all of which may enhance the risk of perinatal oxygen deficiency. Earlier studies reported that perinatal lack of oxygen is associated with DCD.^5^ Additionally, postterm birth can increase the risks of fewer nutrients and less oxygen offered to a fetus larger than normal size by a postterm placenta,^37^ which may be related to atypical motor development. The underlying causes of DCD among children who are born post term should be explored in future research.

Our results also showed that the association between gestational age (children born early term and post term) and suspected DCD did not persist in younger children (aged 3 years). Although there is some debate in the literature about the variability of motor performance,^68^ it has been reported that typical motor development is characterized by variation and the development of adaptive variability, but atypical motor development is characterized by limitations in variation and variability.^69^ Therefore, in early term and postterm birth, the motor performance gap between children with typical and atypical development may become evident as children grow older. Early identification of those at risk of later DCD can provide an important opportunity for early intervention.^70^

### Limitations

Our study had several limitations. In our study, the LDCDQ was used to measure motor performance. The LDCDQ was specifically designed to identify preschoolers at risk of DCD. Although previous studies have shown that the LDCDQ has relatively high sensitivity and specificity,^42,43,71^ there are potential limitations as it is a very short questionnaire. In particular, considering the large variability of motor performance of preschoolers,^37^ the motor capability measured by LDCDQ may not reflect the complete motor profile of young children. Additionally, because this was a retrospective cohort study, our research results cannot be used to identify causal relationships among variables. Possible recall bias may also exist in a retrospective cohort study. Further research is needed to explain the mechanisms linking early and postterm birth to DCD.

## Conclusions

Results of this cohort study suggest that, in addition to children born preterm, children born early term and post term also have an increased risk of DCD. Although the absolute risks of early term and postterm birth were lower than those of preterm birth, children born early term and post term should be monitored more carefully than full-term children; this can be accomplished with long-term follow-up evaluations. Our results may serve to remind clinical professionals, parents, and teachers to not neglect the long-term neurodevelopmental risks of children who are born in the early-term and postterm gestational period. The results of this study may inform clinical professionals when considering the optimal timing of birth during the full-term period. This consideration seems particularly relevant in an era when rates of cesarean delivery are high and induction of labor is common,^19,20^ and those interventions of elective birth should only be recommended if the risk of continuing the pregnancy (postterm birth) is higher than the risk of delivery.^72^

## Notes

Supplement. **eFigure.** The Association of Gestational Age With LDCDQ Scores in Preschoolers When Adjusting for Child, Family, and Maternal Health Characteristics by (A) Sex and (B) Age **eTable 1.** The LDCDQ Scores by Different Gestational Weeks **eTable 2.** The Association Between Gestational Age and LDCDQ Scores in Preschoolers
