# Supplementary material for: Association of Gestational Age at Birth With Subsequent Suspected Developmental Coordination Disorder in Early Childhood in China
Source: JAMA Netw Open. 2021 Dec 14;4(12):e2137581. doi: 10.1001/jamanetworkopen.2021.37581 (PMC8672235; doi:10.1001/jamanetworkopen.2021.37581)
Supplement: Supplement. — eFigure. The Association of Gestational Age With LDCDQ Scores in Preschoolers When Adjusting for Child, Family, and Maternal Health Characteristics by (A) Sex and (B) Age eTable 1. The LDCDQ Scores by Different Gestational Weeks eTable 2. The Association Between Gestational Age and LDCDQ Scores in Preschoolers [file jamanetwopen-e2137581-s001.pdf]

Supplementary Online Content

Hua J, Barnett AL, Williams GJ, et al. Association of gestational age at birth with subsequent suspected developmental coordination disorder in early childhood in China. *JAMA Netw Open*. 2021;4(12):e2137581.
doi:10.1001/jamanetworkopen.2021.37581

eFigure. The Association of Gestational Age With LDCDQ Scores in Preschoolers When Adjusting for Child, Family, and Maternal Health Characteristics by (A) Sex and (B) Age

eTable 1. The LDCDQ Scores by Different Gestational Weeks

eTable 2. The Association Between Gestational Age and LDCDQ Scores in Preschoolers

This supplementary material has been provided by the authors to give readers additional information about their work.

eFigure. The Association of Gestational Age With LDCDQ Scores in Preschoolers When Adjusting for Child, Family, and Maternal Health Characteristics by (A) Sex and (B) Age (n=152,433)

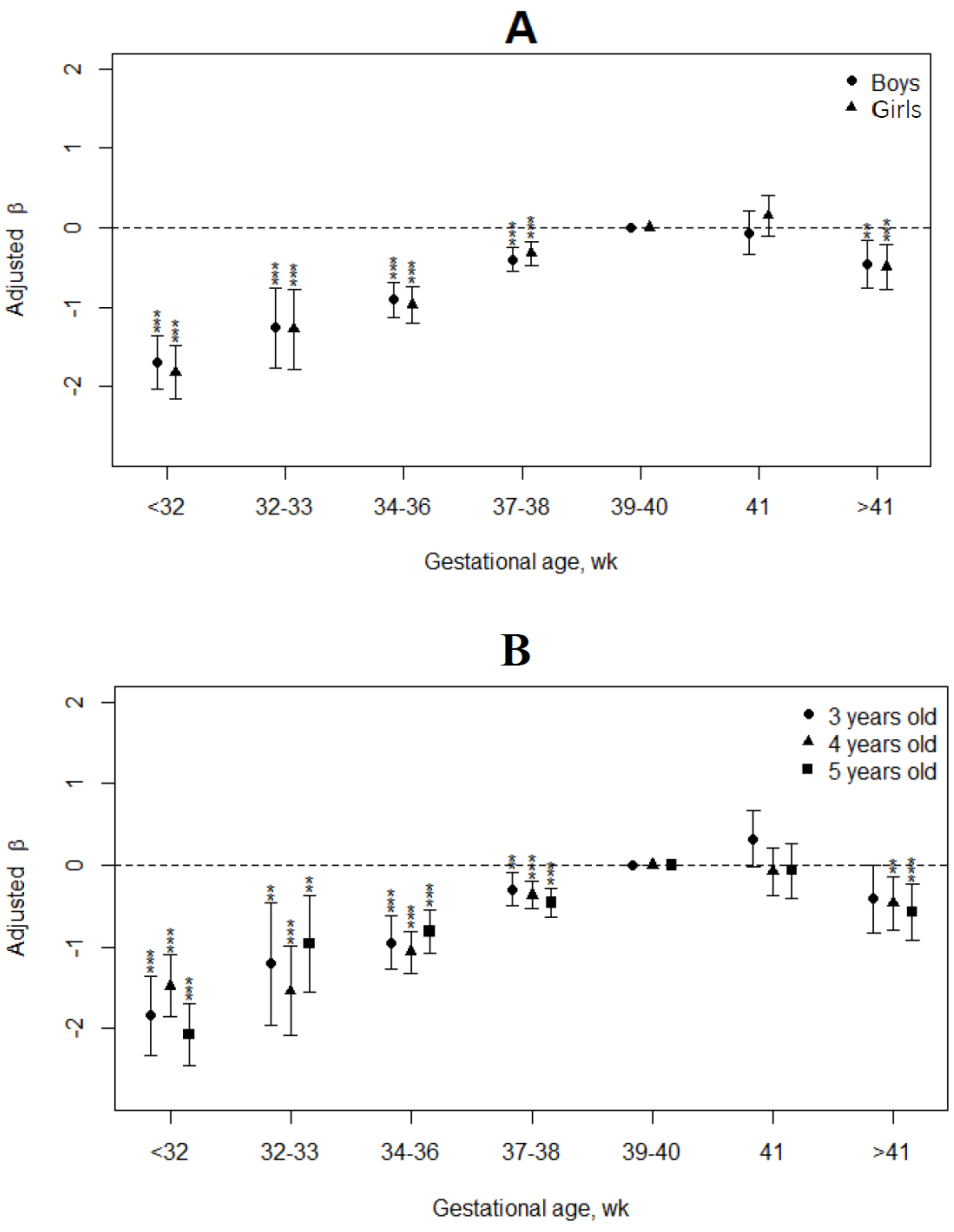

***$p<0.05$, **$p<0.01$, $p<0.001$**

© 2021 Hua J et al. *JAMA Network Open.*

eTable 1. The LDCDQ Scores by Different Gestational Weeks

(n=152,433)

**Gestational weeks**	**Total score**	**Sub-scores of LDCDQ Mean (Standard Deviation)**		
		Motor control	Writing /Fine motor	General coordination
Total children	67.695 (8.94)	22.884(3.11)	22.669 (3.16)	22.1409 (3.23)
<32(very preterm)	65.626 (10.67)	22.140(3.73)	21.983 (3.75)	21.5032 (3.74)
32-33(moderately preterm)	66.235 (10.07)	22.361(3.55)	22.227 (3.54)	21.6469 (3.54)
34-36(late preterm)	66.602(9.53)	22.520 (3.34)	22.316 (3.38)	21.7662 (3.39)
37-38(early-term)	67.559 (9.00)	22.849 (3.12)	22.615 (3.18)	22.0952 (3.25)
39-40(completely full term)	68.125 (8.59)	23.026 (2.98)	22.813(3.04)	22.2865 (3.13)
41(late-term)	68.151 (8.72)	23.052 (3.01)	22.823(3.10)	22.2748 (3.15)
>41(post-term)	67.293 (9.15)	22.755(3.17)	22.544 (3.25)	21.9932 (3.29)

© 2021 Hua J et al. *JAMA Network Open.*

eTable 2. The Association Between Gestational Age and LDCDQ Scores in Preschoolers **(n=152,433)**

**Gestational weeks**	Total score		Motor control		Writing/Fine motor		General coordination	
	Crude β (95% CI)	Adjusted β[a] (95% CI)	Adjusted β (95% CI)	Adjusted β[a] (95% CI)	Crude β (95% CI)	Adjusted β[a] (95% CI)	Adjusted β (95% CI)	Adjusted β[a] (95% CI)
<32(very preterm)	-2.19 (-2.43, -1.95) *p*<0.001	-1.74 (-1.98, -1.50) *p*<0.001	-0.79 (-0.87, -0.70) *p*<0.001	-0.65 (-0.73, -0.56) *p*<0.001	-0.74 (-0.82, -0.65) *p*<0.001	-0.60 (-0.68, -0.51) *p*<0.001	-0.67 (-0.76, -0.58) *p*<0.001	-0.51 (-0.59, -0.42) *p*<0.001
32-33(moderately preterm)	-1.70 (-2.07, -1.34) *p*<0.001	-1.24 (-1.60, -0.89) *p*<0.001	-0.60 (-0.73, -0.47) *p*<0.001	-0.46 (-0.58, -0.33) *p*<0.001	-0.53 (-0.66, -0.41) *p*<.001	-0.39 (-0.52, -0.27) *p*<0.001	-0.57 (-0.71, -0.44) *p*<0.001	-0.41 (-0.54, -0.28) *p*<0.001
34-36(late preterm)	-1.30 (-1.46, -1.13) *p*<0.001	-0.92 (-1.08, -0.76) *p*<0.001	-0.43 (-0.49, -0.38) *p*<0.001	-0.32 (-0.38, -0.27) *p*<0.001	-0.43 (-0.49, -0.37) *p*<0.001	-0.31 (-0.36, -0.25) *p*<0.001	-0.44 (-0.50, -0.38) *p*<0.001	-0.30 (-0.36, -0.24) *p*<0.001
37-38(early-term)	-0.50 (-0.61, -0.40) *p*<0.001	-0.36 (-0.46, -0.25) *p*<0.001	-0.16 (-0.20, -0.12) *p*<0.001	-0.12 (-0.16, -0.09) *p*<0.001	-0.18 (-0.21, -0.14) *p*<0.001	-0.12 (-0.16, -0.08) *p*<0.001	-0.17 (-0.21, -0.13) *p*<0.001	-0.12 (-0.16, -0.08) *p*<0.001
39-40(completely full-term)	Reference	Reference	Reference	Reference	Reference	Reference	Reference	Reference
41(late-term)	0.02 (-0.17,0.22)	0.06 (-0.13, 0.24)	0.03 (-0.04, 0.09)	0.04 (-0.03, 0.10)	0.02 (-0.05, 0.08)	0.02 (-0.04, 0.09)	-0.02 (-0.09, 0.05)	-0.01 (-0.07, 0.06)
>41(post-term)	-0.66 (-0.87, -0.45) *p*<0.001	-0.47 (-0.67, -0.26) *p*<0.001	-0.22 (-0.29, -0.14) *p*<0.001	-0.15 (-0.22, -0.08) *p*<0.001	-0.22 (-0.29, -0.14) *p*<0.001	-0.17 (-0.24, -0.09) *p*<0.001	-0.23 (-0.31, -0.16) *p*<0.001	-0.16 (-0.23, -0.08) *p*<0.001

[a]LDCDQ refers to the little Developmental Coordination Disorder Questionnaire.

[b]Adjusting for clusters (to control the unmeasured factors in kindergarten environment), child and family characteristics and maternal health during pregnancy.

© 2021 Hua J et al. *JAMA Network Open.*
